# Characterization of the mitochondrial genome of the pathogenic fungus *Scytalidium auriculariicola* (Leotiomycetes) and insights into its phylogenetics

**DOI:** 10.1038/s41598-019-53941-5

**Published:** 2019-11-25

**Authors:** Cheng Chen, Qiang Li, Rongtao Fu, Jian Wang, Chuan Xiong, Zhonghan Fan, Rongping Hu, Hong Zhang, Daihua Lu

**Affiliations:** 1 Institute of plant protection, Sichuan Academy of Agricultural Sciences, Chengdu, 610066 Sichuan P.R. China; 2 Key Laboratory of Integrated Pest Management on Crops in Southwest, Ministry of Agriculture, Chengdu, 610066 Sichuan P.R. China; 3 College of Pharmacy and Biological Engineering, Chengdu University, Chengdu, 610106 Sichuan P.R. China; 4 Biotechnology and Nuclear Technology Research Institute, Sichuan Academy of Agricultural Sciences, Chengdu, 610061 Sichuan P.R. China; 5 Present Address: Sichuan Academy of Agricultural Sciences, 20 # Jingjusi Rd, Chengdu, 610066 Sichuan P.R. China

**Keywords:** Evolution, Microbiology

## Abstract

*Scytalidium auriculariicola* is the causative pathogen of slippery scar disease in the cultivated cloud ear fungus, *Auricularia polytricha*. In the present study, the mitogenome of *S. auriculariicola* was sequenced and assembled by next-generation sequencing technology. The circular mitogenome is 96,857 bp long and contains 56 protein-coding genes, 2 ribosomal RNA genes, and 30 transfer RNA genes (tRNAs). The high frequency of A and T used in codons contributed to the high AT content (73.70%) of the *S. auriculariicola* mitogenome. Comparative analysis indicated that the base composition and the number of introns and protein-coding genes in the *S. auriculariicola* mitogenome varied from that of other Leotiomycetes mitogenomes, including a uniquely positive AT skew. Five distinct groups were found in the gene arrangements of Leotiomycetes. Phylogenetic analyses based on combined gene datasets (15 protein-coding genes) yielded well-supported (BPP = 1) topologies. A single-gene phylogenetic tree indicated that the *nad4* gene may be useful as a molecular marker to analyze the phylogenetic relationships of Leotiomycetes species. This study is the first report on the mitochondrial genome of the genus *Scytalidium*, and it will contribute to our understanding of the population genetics and evolution of *S. auriculariicola* and related species.

## Introduction

*Scytalidium auriculariicola* is the causative pathogen of slippery scar on the cloud ear fungus *Auricularia polytricha* (Mont.) Sacc^1^. The pathogen only infects the mycelia of *A. polytricha* and not the fruiting body^2^. Slippery scar first appeared in cultivated *A. polytricha* in Sichuan Province in the 1990s, with the infection rate being over 40%^2^. The identity of the pathogen of slippery scar has been controversial and has undergone a process of re-identification and re-establishment. Sun *et al*.^2^ initially identified the pathogen as the Ascomycete *S. lignicola* according to Koch’s postulates, morphological observations, rDNA-internal transcribed spacer (ITS), and 18S sequence analysis. Peng *et al*.^1^ further investigated the causative pathogen of this disease by morphological observations, *in vivo* pathogenicity tests, and molecular evidence from rRNA ITS and RNA polymerase II subunit (*RPB2)* sequences. Their results showed that the pathogen is a new species in the *Scytalidium* genus differing from *S. lignicola*, which they named *S. auriculariicola*^1^. Peng *et al*.^1^ also pointed out that *S. auriculariicola* strains from diseased *A. polytricha* mycelium in China consist of two clonal groups. Previously, ITS rDNA sequences, 18S rDNA sequences, RPB2 genes, and ribosomal large subunit (LSU) genes have been used as molecular markers to determine species relationships between *Scytalidium* and other species within the Leotiomycetes^1,3–5^. However, limited genetic information has prevented a comprehensive understanding of phylogenetic relationships between *Scytalidium* and its related species^6^. Thus, more available molecular markers are needed to assess the relationships between *Scytalidium* and other taxa within the Leotiomycetes, especially between the important *S. auriculariicola* and its related species.

Mitochondria are organelles necessary for the life of eukaryotic cells, and their dysfunction is associated with disease, the aging process, development, and various biological traits^7,8^. Mitochondria are thought to originate from symbiotic bacteria and supply most of the energy for eukaryotes^9^. Mitochondrial genomes are independent from nuclear genomes and have maternal inheritance, a high copy number, larger sequence length than the rRNA gene, which has made them widely used as a tool for studying evolution, phylogenetics, population genetics, and comparative or evolutionary genomics^10,11^. Mitochondrial gene rearrangements and the secondary structure of tRNAs are also widely used for deep-level phylogenetic studies in eukaryotes^12–14^. However, the mitogenomes of fungi are not as well studied as those of animals and plants^15^. The limited available studies have shown that most fungal mitochondrial genomes are circular, with the exception of very few species with linear mitogenomes^16,17^. The size of fungal mitogenomes varies greatly, ranging from 18.84 kb (*Hanseniaspora uvarum*) to 235.85 kb^18^. Fungal mitochondrial DNA usually contains 14 conserved protein-coding genes (*atp6, atp8, atp9, cob, cox1, cox2, cox3, nad1, nad2, nad3, nad4, nad4L, nad5*, and *nad6*), 1 ribosomal protein S3 gene (*rps3*), 2 ribosomal RNA genes (*rnl* and *rns*), and a relatively constant set of tRNA genes^19^. Fungal mitogenomes sometimes include homing endonuclease genes, plasmid-derived genes, genes transferred from the nuclear genome, and genes of unknown function. The number and variation of mitochondrial genes, genome rearrangement, and the presence or absence of large intronic and intergenic sequences all contribute to the considerable variation in gene content, structure, and size of fungal mitogenomes. To date, no mitochondrial genomes are available in the *Scytalidium*, and only 16 species from the Leotiomycetes class, including *Cairneyella variabilis*, *Marssonina brunnea*, *Phialocephala subalpina*, 2 *Glarea lozoyensis* subspecies, 4 *Pseudogymnoascus* species, 4 *Rhynchosporium* species, 2 *Sclerotinia* species, and *Botrytis cinera* (teleomorph *Botryotinia fuckeliana*), have been sequenced^20^ (https://www.ncbi.nlm.nih.gov/genome/browse#!/organelles/Leotiomycetes). More mitogenomes are needed, especially from the *Scytalidium* genus, which contains multiple pathogens, to reveal species and genus-level phylogenetic relationships in Leotiomycetes.

In the present study, the pathogen *S. auriculariicola* was isolated, identified, and sequenced. We assembled and annotated the complete mitochondrial genome of *S. auriculariicola* and assessed its gene content, tRNA structure, and genome organization. The mitochondrial genome size, base composition, number of protein-coding genes, tRNA genes, and gene arrangement of *S. auriculariicola* and previously sequenced species within Leotiomycetes were compared. We then analyzed the phylogenetic relationships among various Leotiomycetes species based on mitochondrial genes. The mitochondrial genome of *S. auriculariicola* will allow further investigations into the taxonomy, phylogenetics, conservation genetics, and evolutionary biology of this important genus as well as other closely related species.

## Results

### Protein coding genes, rRNA genes, tRNA genes, intergenic regions, and codon analyses

**Figure 1:**
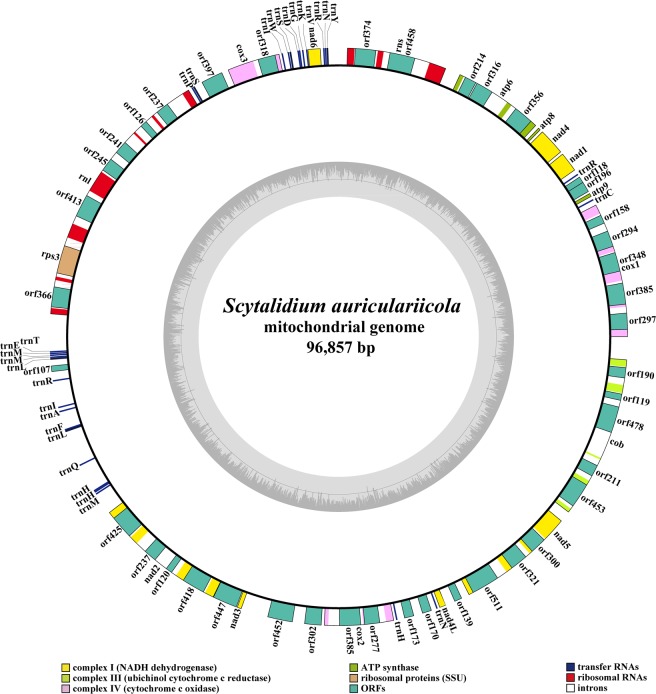
Circular map of the *Scytalidium auriculariicola* mitochondrial genome. Various genes are represented with different color blocks. The mitochondrial circular map was drawn using the OGDRAW software^58^.

The mitogenome of *S. auriculariicola* is composed of a circular DNA molecule of 96,857 bp (Fig. 1). The GC content of the *S. auriculariicola* mitogenome is 26.30%. The AT skew and GC skew were both positive in the *S. auriculariicola* mitogenome (Table 1). A total of 56 protein-coding genes were identified in the mitogenome of *S. auriculariicola*, including 15 core mitochondrial genes (*atp6, atp8, atp9, cob, cox1, cox2, cox3, nad1, nad2, nad3, nad4, nad4L, nad5, nad6*, and *rps3*) and 41 ORFs (Table S1). Among these ORFs, we found 32 ORFs located in introns and the remaining 9 ORFs were regarded as free-standing. Twenty-one out of the 32 intronic ORFs were predicted to encode proteins that exhibit similarities to the homing endonucleases from the LAGLIDADG family. Eight ORFs are similar to homing endonucleases in the family GIY-YIG, and 3 ORFs were predicted to encode hypothetical proteins. We also found seven free-standing ORFs that were predicted to encode proteins that exhibit similarities to homing endonucleases in the LAGLIDADG (four ORFs) and GIY-YIG (three ORFs) families. In addition, two free-standing ORFs were predicted to encode hypothetical proteins (Table S1). All of the 56 detected mitochondrial genes were located on the sense strand.

**Table 1:** Comparison of Leotiomycetes mitogenomes.

Item	Accession number	Genome size (bp)	GC content (%)	AT skew	GC skew	conserved PCGs	free-standing ORFs	No. of introns	Intronic ORFs	No. of tRNAs
*Scytalidium auriculariicola*	MK111108	96,857	26.30	0.030	0.127	14	9	27	32	30
*Cairneyella variabilis*	NC_029759	27,186	26.27	−0.043	0.133	14	1	1	1	29
*Glarea lozoyensis*	NC_031375	45,501	29.76	−0.041	0.081	14	4	5	2	30
*Marssonina brunnea*	NC_015991	70,379	29.34	−0.009	0.068	14	8	18	13	28
*Phialocephala subalpina*	NC_015789	43,742	27.95	−0.029	0.091	14	9	0	0	27
*Pseudogymnoascus destructans*	NC_033907	32,181	28.53	−0.048	0.116	13	3	2	2	28
*Pseudogymnoascus pannorum*	NC_027422	26,918	28.10	−0.063	0.113	13	1	1	1	27
*Pseudogymnoascus_*sp*._*04NY16	CM004376	32,146	28.66	−0.050	0.118	13	4	2	2	28
*Pseudogymnoascus_*sp*._*BL308	CM004375	32148	28.55	−0.045	0.112	13	4	2	2	29
*Rhynchosporium agropyri*	NC_023125	68,904	29.36	−0.006	0.066	14	9	19	25	29
*Rhynchosporium commune*	NC_023126	69,581	29.40	−0.006	0.068	14	9	18	25	29
*Rhynchosporium orthosporum*	NC_023127	49,539	28.80	−0.028	0.086	14	9	3	2	32
*Rhynchosporium secalis*	NC_023128	68,729	29.33	−0.007	0.069	14	8	18	25	29
*Sclerotinia borealis*	NC_025200	203,051	32.01	−0.001	0.084	15	18	61	62	31
*Sclerotinia sclerotiorum*	NC_035155	128,852	30.90	−0.007	0.103	14	21	36	32	35
*Botrytis cinerea*	Broad Institute	82,212	29.90	−0.008	0.100	14	7	22	20	33

**Figure 2:**
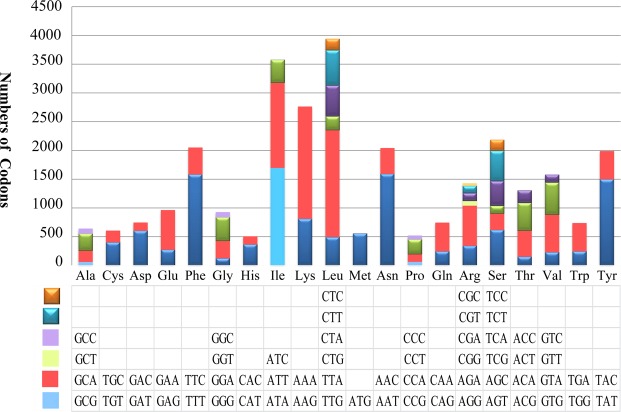
Codon usage in the *Scytalidium auriculariicola* mitochondrial genome. Codon families are plotted on the X axis and represented by different color patches. Frequency of codon usage is plotted on the Y axis.

The mitogenome of *S. auriculariicola* contains two rRNA genes, a large subunit ribosomal RNA gene (*rnl*), and a small subunit ribosomal RNA gene (*rns*). A total of 30 tRNA genes were detected. The length of individual tRNAs ranges from 71 to 86 bp, mainly due to the different sizes of the extra arms (Fig. 2). The main cluster of tRNA genes (16 tRNAs) is located around the *rnl* gene. The other two clusters, including ten and two tRNA genes, are located around the *nad6* and *atp9* genes, respectively. In addition, there are two other tRNA genes located between the *nad4L* and *cox2* genes (Fig. 1). There are mispairings of bases in 27 out of the 30 tRNAs, with a total of 45 base mispairings, all of which are G-U mispairings (Table S2). The *S. auriculariicola* mitogenome contains two tRNAs that code for asparagine with the same anticodons, three tRNAs coding for methionine with the same anticodons, and three tRNAs that code for histidine with the same anticodons. In addition, there are four amino acids that have more than one anticodon coding for them (Table S3).

The mitogenome of *S. auriculariicola* contains three pairs of overlapping genes: orf196/orf118 overlap by 23 nucleotides, *nad4L*/*nad5* and *nad2*/orf447 overlap by 1 bp. In addition, the *S. auriculariicola* mitogenome contains 55 intergenic regions with lengths ranging from 5 to 1986 bp, with a total length of 20,534 bp (Table S1). Intergenic nucleotides account for 21.20% of the mitogenome.

**Figure 3:**
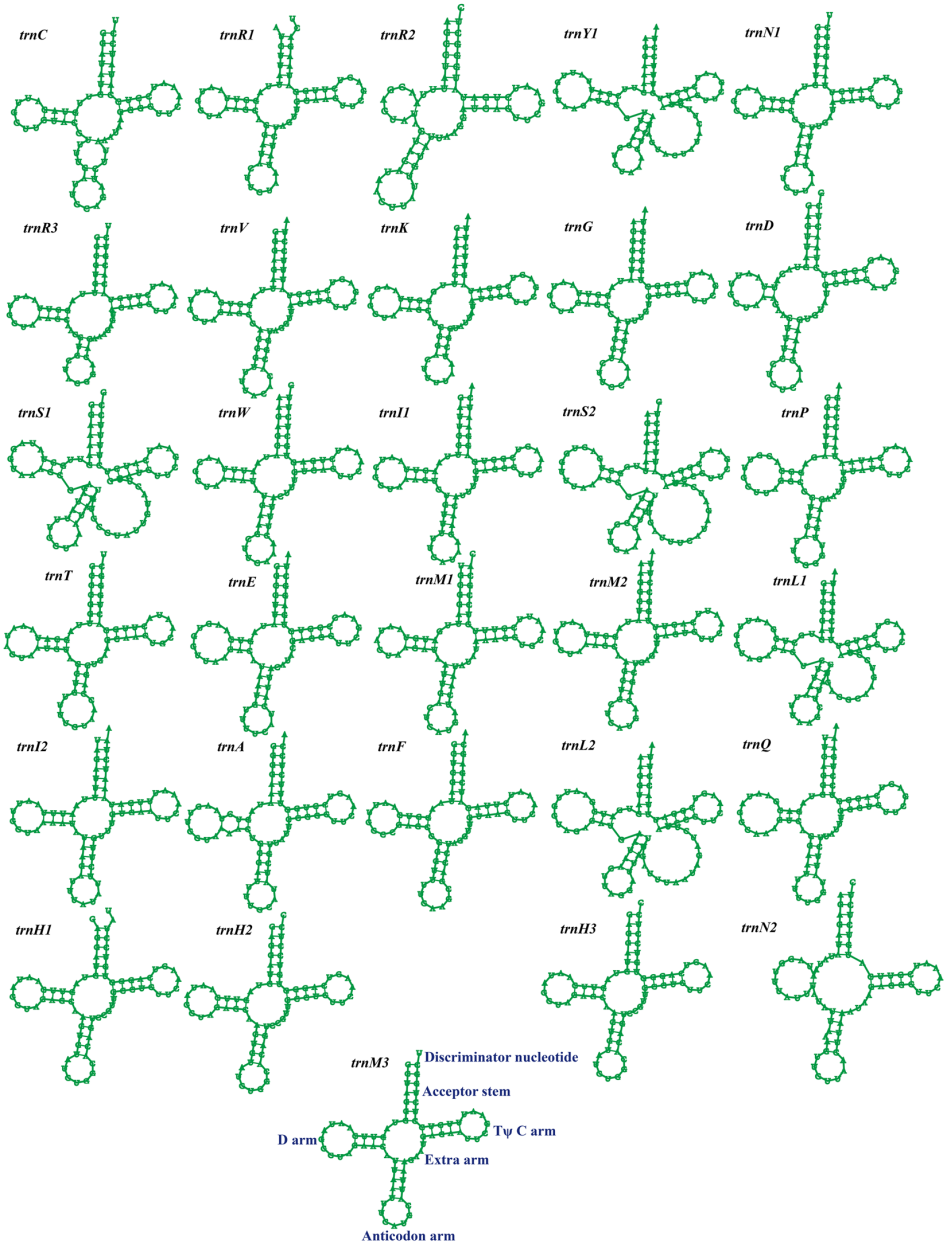
Putative secondary structures of the 30 tRNA genes from *Scytalidium auriculariicola* mitochondrial genome. The tRNAs are labeled with the abbreviations of their corresponding amino acids. The tRNA arms are illustrated as for trnM3. The map of tRNA structures was drawn using the mitos software^69^.

All of the 15 conserved protein-coding genes start with the canonical translation initiation codon ATG except for *cox1* and *nad4*, which start with TTG (Table S4). Five genes (*atp9*, *cox3*, *nad3*, *cox2*, and *rps3*) use TAG as the stop codon while the others use TAA. Codon usage analysis indicated that the most frequently used codons in the *S. auriculariicola* mitogenome are AAA (6.53%, for lysine; Lys), TTA (6.23%, leucine; Leu), ATA (5.68%, for isoleucine; Ile), AAT (5.34%, for asparagine; Asn), TTT (5.31%, for phenylalanine; Phe), and TAT (5.02%, for tyrosine; Tyr) (Fig. 3; Table S5). The high frequency of A and T use in codons contributes to the high AT content (73.70%) of the *S. auriculariicola* mitochondrial genome.

### Repetitive elements analysis

There are 33 repeat regions that were identified by a BLASTn search of the *S. auriculariicola* mitogenome against itself. The size of the repeats ranges from 39 to 610 bp; 19 repeat regions are over 100 bp, and 10 are over 200 bp. The similarities of these repeated sequences are between 74.81% and 100%. The longest repeat regions are located in the intergenic region between *nad2* and orf173. The repeat sequences account for 5.65% of the entire *S. auriculariicola* mitogenome (Table S6).

We detected 26 tandem repeats in the mitogenome of *S. auriculariicola*. The length of the tandem repeats ranges from 6 to 77 bp. Copy numbers of each tandem repeat were between 2.0 and 16.5. The longest tandem sequence occurs with two copies. The tandem sequences account for 1.58% of the entire mitogenome (Table S7). We identified 47 forward, 1 palindromic, and 2 reverse repeats in the mitogenome of *S. auriculariicola*, accounting for 3.70% of the entire mitogenome (Table S8).

### Comparative genome analysis

The size of *S. auriculariicola* mitogenome is the third largest genome among the 16 mitogenomes in the Leotiomycetes (Table 1), being only smaller than those of *Sclerotinia borealis* and *Sclerotinia sclerotiorum*, which belong to the family Sclerotiniaceae, order Helotiales. The GC content of the *S. auriculariicola* mitogenome is very low (26.30%) and is only higher than that of *C. variabilis* (26.27%). Both the AT skew and GC skew of the *S. auriculariicola* mitogenome are positive, while the AT skew is negative and GC skew positive in the other mitogenomes from the Leotiomycetes. The number of protein-coding genes and introns is closely related to the size of mitogenomes. The *S. borealis* with the largest mitogenome has 95 protein-coding genes and 61 introns, while the *P. pannorum* with the smallest mitogenome has only 15 protein-coding genes and one intron. The number of tRNA genes in the mitogenomes of Leotiomycetes varies from 27 to 35 (Table 1).

The composition of Leotiomycetes mitogenomes is shown in Table 2. The size of RNA genes and conserved protein-coding genes is relatively conservative between different species. The size of introns and intergenic regions is found closely related to the size variations of mitogenomes. Free-standing ORFs also contribute to the size variation of Leotiomycetes mitogenomes, which varies greatly between different species. The mitogenome of *S. sclerotiorum* contained the largest size of free-standing ORFs, followed by *S. borealis*, *R. orthosporum* and *P. subalpina*. The mitogenome of *S. borealis* contains the largest repeated sequences (exceeding 20 kb), while *C. variabilis* mitogenome contains the smallest (only 30 bp).

**Table 2:** The size of different mitochondrial regions and their proportion to the whole mitogenome.

ID	Mitogenome	RNA genes		Introns		Conserved PCGs		Free-standing ORFs		Intergenic regions		Repeated sequences	
	size (bp)	size (bp)	Proportion	size (bp)	proportion	size (bp)	proportion	size (bp)	proportion	size (bp)	proportion	size (bp)	proportion
Sbo	203051	7000	**3.45**	125696	**61.90**	13935	**6.86**	10461	**5.15**	45959	**22.63**	23122	**11.39**
Ssc	128852	7016	**5.45**	53723	**41.69**	12757	**9.90**	12690	**9.85**	42666	**33.11**	12321	**9.56**
Sau	96857	6865	**7.09**	48702	**50.28**	13668	**14.11**	7113	**7.34**	20509	**21.17**	5470	**5.65**
Bci	82212	7343	**8.93**	31793	**38.67**	13242	**16.11**	6351	**7.73**	23483	**28.56**	4723	**5.74**
Mbr	70379	6916	**9.83**	19289	**27.41**	15098	**21.45**	6246	**8.87**	22830	**32.44**	6402	**9.10**
Rco	69581	7073	**10.17**	27385	**39.36**	14118	**20.29**	6633	**9.53**	14372	**20.66**	3551	**5.10**
Rag	68904	7074	**10.27**	28435	**41.27**	13529	**19.63**	6024	**8.74**	13842	**20.09**	2694	**3.91**
Rse	68729	7073	**10.29**	27385	**39.84**	15099	**21.97**	6255	**9.10**	12917	**18.79**	3466	**5.04**
Ror	49539	7310	**14.76**	4016	**8.11**	13419	**27.09**	9177	**18.52**	15617	**31.52**	7363	**14.86**
Glo	45501	7432	**16.33**	5525	**12.14**	15456	**33.97**	2886	**6.34**	14202	**31.21**	5726	**12.58**
Psu	43742	7411	**16.94**	0	**0.00**	13938	**31.86**	8217	**18.79**	14176	**32.41**	2838	**6.49**
Pde	32181	6495	**20.18**	3334	**10.36**	12681	**39.41**	3429	**10.66**	6242	**19.40**	1144	**3.55**
Psp.1	32148	6564	**20.42**	3332	**10.36**	12488	**38.85**	2967	**9.23**	6797	**21.14**	1112	**3.46**
Psp.2	32146	6493	**20.20**	3332	**10.37**	12359	**38.45**	2925	**9.10**	7037	**21.89**	1218	**3.79**
Cva	27186	7147	**26.29**	1905	**7.01**	13176	**48.47**	753	**2.77**	4205	**15.47**	30	**0.11**
Ppa	26918	6407	**23.80**	1999	**7.43**	12621	**46.89**	363	**1.35**	5528	**20.54**	742	**2.76**

Species information in this table can be found in Supplementary Table S9.

### Gene rearrangements

**Figure 4:**
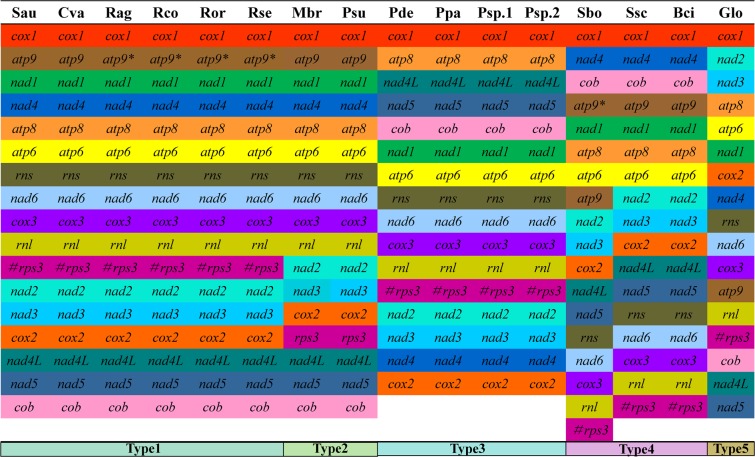
Comparison of gene order across 16 Leotiomycetes mitogenomes. Cva, *Cairneyella variabilis* (NC_029759); Glo, *Glarea lozoyensis* (NC_031375); Mbr, *Marssonina brunnea* f. sp. Multigermtubi (NC_015991); Psu, *Phialocephala subalpine* (NC_015789); Pde, *Pseudogymnoascus destructans* (NC_033907); Ppa, *Pseudogymnoascus pannorum* (NC_027422); Psp1, *Pseudogymnoascus*_sp._04NY16 (CM004376); Psp2, *Pseudogymnoascus*_sp._BL308 (CM004375); Rag, *Rhynchosporium agropyri* (NC_023125); Rco, *Rhynchosporium commune* (NC_023126); Ror, *Rhynchosporium orthosporum* (NC_023127); Rse, *Rhynchosporium secalis* (NC_023128); Sbo, *Sclerotinia borealis* (NC_025200)*;* Ssc, *Sclerotinia sclerotiorum* 1980 UF-70 (NC_035155); Bci, *Botrytis cinerea* (Broad Institute); Sau, *Scytalidium auriculariicola* (MK111108). The # symbol indicates the *rps3* gene is located in the intron of the *rnl* gene.

Five different groups of gene arrangement were detected in the 16 Leotiomycetes mitogenomes (Fig. 4). The results indicated that Leotiomycetes have undergone large-scale gene rearrangements in their mitogenomes over the course of evolution. The gene order of the *S. auriculariicola* mitogenome is identical to that of *C. variabilis*, which belongs to the Helotiaceae, order Helotiales. However, large-scale gene rearrangements were observed in the *G. lozoyensis* mitogenome, another species of the Helotiaceae. The mitochondrial gene order of four *Rhynchosporium* species, *R. agropyri*, *R. commune*, *R. orthosporum*, and *R. secalis*, is the same as that of *S. auriculariicola*. However, the *atp9* genes of *Rhynchosporium* sp. are likely dysfunctional due to a premature stop codon in a conserved domain^21^. The mitochondrial gene order of *S. auriculariicola* is also similar to those of *M. brunnea* and *P. subalpine*, with the position of the *rps3* gene in their mitogenomes differing. In the *Pseudogymnoascus* genus, we observed an identical mitochondrial gene order between the four *Pseudogymnoascus* species, *P. destructans, P. pannorum, P*. sp.04NY16, and *P*. sp.BL308. In the Sclerotiniaceae family, the order of mitochondrial genes of three Sclerotiniaceae species, *S. sclerotiorum*, *S. borealia*, and *B. cinerea*, is identical, except for minor differences (the location of *atp9*) in the *S. borealia* mitogenome due to replication events of *atp9* genes. In addition, large-scale gene rearrangements were observed between the mitogenomes of *S. auriculariicola*, the Sclerotiniaceae, and *Pseudogymnoascus*.

Compared with the arrangement of *rps3* genes in type 1, the *rps3* genes in the gene arrangement type 2 become free-standing ORFs, and are transferred to the downstream of the *cox2* gene. The gene arrangement type 3 is characterized by the loss of *atp9* gene, and the relocation of *nad4, cox2, atp8, nad4L, nad5, cob*, and *nad1* genes. For the gene arrangement type 4, the *rns, nad6, cox3, rnl* and *rps3* genes are transferred to the downstream of *nad5* gene; *nad4* and *cob* genes are transferred to the upstream of the *atp9* gene. The type5 involves multiple gene repositions, including *atp9, cox2, cox3, nad1, nad2, nad3*, and *nad4* genes.

**Figure 5:**
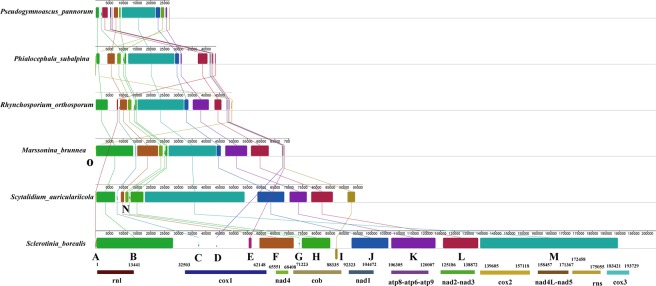
Mitogenome synteny among six Leotiomycetes species. Synteny analyses were generated in Mauve 2.4.0. Fifteen homologous regions were identified among the six mitogenomes. The sizes and relative positions of the homologous fragments varied across the mitogenomes.

Synteny analysis indicated that the six Leotiomycetes mitogenomes can be divided into fifteen homologous regions, where the sizes and relative positions of these regions are highly variable (Fig. 5). Nine of the fifteen homologous regions were detected in all six mitogenomes. The *R. orthosporum* mitogenome has all 15 homologous regions while the other mitogenomes lack one to three homologous regions. *M. brunnea* lacks the homologous region ‘A’, *S. borealia* lacks the homologous regions ‘N’ and ‘O’, and *S. auriculariicola* lacks the homologous regions ‘D’, ‘E’, and ‘O’.

### Phylogenetic analysis

**Figure 6:**
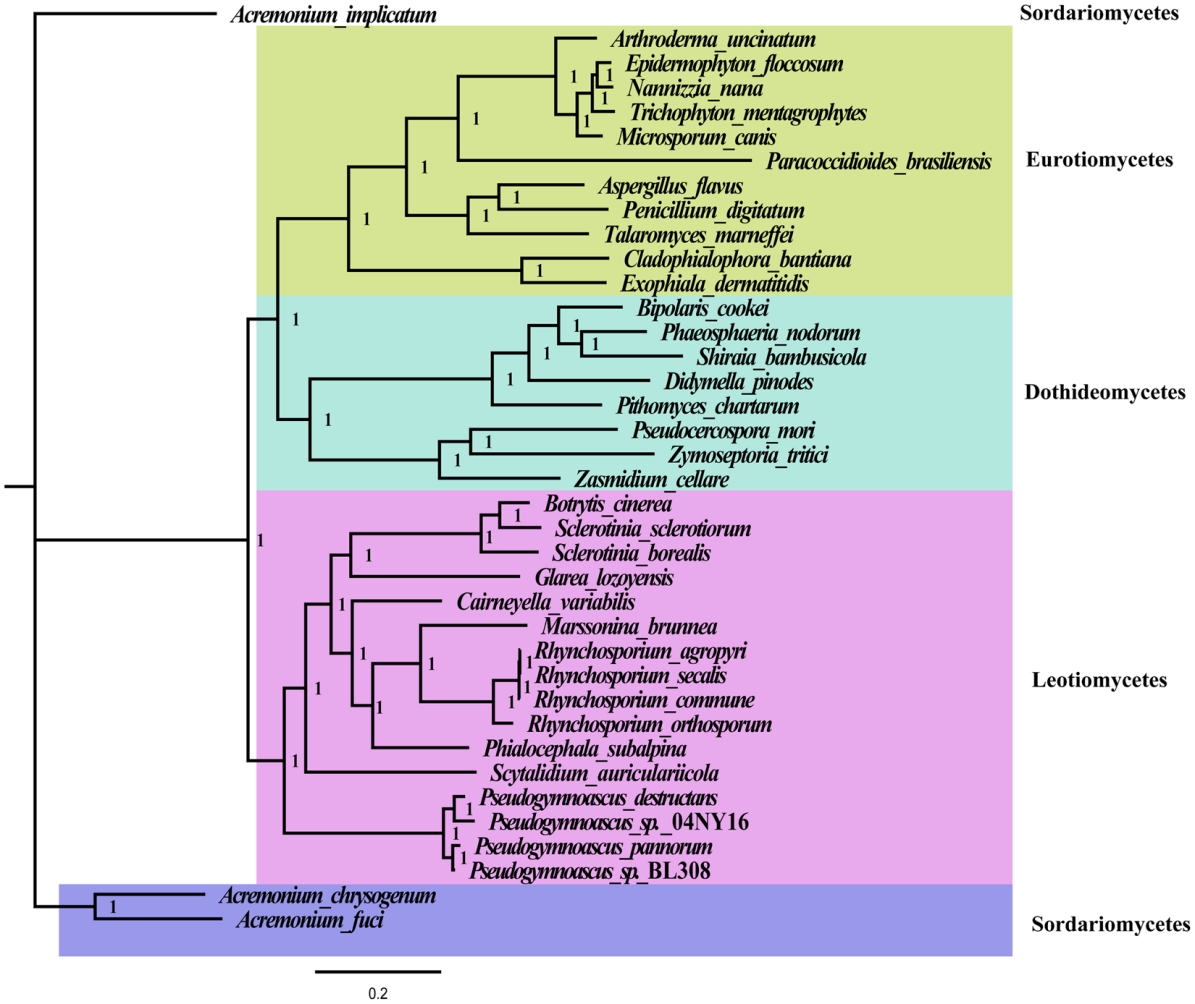
The phylogenetic tree was calculated from the multiple sequence alignment of the combined mitochondrial gene set (15 PCGs) of 38 fungal species. Topology was inferred using Bayesian method. Species analysed are shown in Table S9. The phylogenetic tree was drawn using the Figtree v1.4.3 software (https://mac.softpedia.com/get/Graphics/FigTree.shtml).

We used the Bayesian inference method to establish the phylogenetic relationships of 16 species within Leotiomycetes, 8 species from Dothideomycetes, and 11 species from Eurotiomycetes based on the combined mitochondrial gene set (15 typical protein-coding genes) using three *Acremonium* species, *A. chrysogenum*, *A. fuci*, and *A. implicatum*, as outgroups. The best-fit evolutionary model for the phylogenetic analysis is “GTR + I + G”. We obtained a stable evolutionary tree topology (Fig. 6) with all of the recovered clades well supported (Bayesian posterior probability (BPP) = 1). Based on the phylogenetic analysis, the 38 Pezizomycotina species could be divided into four major clades corresponding to the classes Sordariomycetes, Eurotiomycetes, Dothideomycetes, and Leotiomycetes^20^. The 16 Leotiomycetes species were divided into three clades in the phylogenetic tree. The genus *Pseudogymnoascus* containing four species, *P. pannorum*, *P. destructans*, *P*. sp.04NY16, and *P*. sp.BL308, is the first clade. *S. auriculariicola* is the second single clade. The third clade contains 11 fungal species within the order Helotiales. In the order Helotiales, the relationships of the three families were consistently recovered as (Sclerotiniaceae + (Helotiaceae + (Dermateaceae)).

Single-gene tree topologies varied (Fig. S1), which indicates incongruent phylogenetic signals among different genes. However, the BI phylogenies based on the *nad4* gene were consistent with the all-protein-coding genes phylogeny. Therefore, the *nad4* gene may be a useful barcode sequence for species identification and phylogenetic analysis within the Leotiomycetes.

The phylogenetic analysis of nuclear multi-locus polygenes yielded evolutionary trees different from that of mitochondrial genes (Fig. S2). The BPP value of the nuclear multi-locus phylogenetic tree is between 0.43 and 1. *Pseudocercospora mori*, belonging to the Dothideomycetes class, is incorrectly clustered into the Eurotiomycetes class in the nuclear multi-locus phylogenetic tree. *Exophiala dermatitidis* and *Cladophialophora bantiana*,both belonging to the Eurotiomycetes class, are also incorrectly clustered into the Dothideomycetes class. In addition, *M. brunnea*, belonging to the order of Helotiales, forms an independent clade separated from other Helotiales species. The results further prove that mitochondrial genes are suitable as reliable tools for analyzing phylogenetic relationships of Leotiomycetes species.

## Discussion

Mitochondrial markers have been successfully applied in phylogenetic taxonomy and evolutionary biology due to the advantage of their faster evolution^22,23^. The advent of next-generation sequencing technology has promoted the sequencing of fungal mitochondrial genomes. However, the mitogenomes of fungi are still less well-understood than those of animals and plants^24^. As of July 2019, only 612 mitogenomes of fungi have been reported in the NCBI database, 488 of which belong to the Ascomycetes. However, most species from the Ascomycetes belong to the Saccharomycetes and Sordariomycetes, which account for more than 78%, while the Leotiomycetes account for less than 4%. More mitogenomes belonging to Leotiomycetes are needed to facilitate our understanding of the mitochondrial characteristics and evolution of this group. In the present study, we first sequenced and analyzed the *S. auriculariicola* mitogenome, which is the first reported sequencing of a mitogenome of the *Scytalidium* genus. The mitogenome size of *S. auriculariicola* is larger than that of other Leotiomycetes, with the exception of *S. borealis* and *S. sclerotiorum*^20^. Variability in the length of mitogenomes has been frequently observed in the Leotiomycetes class, which is consistent with the variable mitogenomes in many eukaryotes^25,26^. The largest mitogenome in Leotiomycetes fungi is 203.05 kb^20^, while *P. pannorum* contains the smallest mitogenome at 26.92 kb (Table 1). The *S. borealis* mitogenome has the largest number of introns (61) in the Leotiomycetes, while only one intron was detected in the *P. pannorum* mitogenome. Another species, *C. variabilis*, which contained a mitogenome of less than 30 kb, also had only one intron. Therefore, the number of introns was considered one of the main factors contributing to the mitogenome size variation^27,28^. Interestingly, the intron-free species, *P. subalpina*, has a mitogenome size of 43.74 kb, higher than that of *P. destructans*, *P*. sp.04NY16, and *P*. sp.BL308, which have two introns each. Similarly, the number of introns in the *R. orthosporum* mitogenome is less than that of *G. lozoyensis*, which contains a much smaller mitogenome. Previous studies have shown that the size of fungal mitogenomes is related to the number of introns, intergenic regions, repetitive elements, plasmid-derived regions, and gene transfer events^29,30^. In the present study, we found that the number of free-standing ORFs of the *P. subalpina* mitogenome is higher than that of *P. destructans*, *Pseudogymnoascus* sp.04NY16, and *Pseudogymnoascus* sp.BL308. Furthermore, the number of free-standing ORFs of the *R. orthosporum* mitogenome is more than that of *G. lozoyensis*. The size of free-standing ORFs in *P. subalpina* and *R. orthosporum* mitogenomes accounts for 18.79% and 18.52% of their total mitogenomes, respectively (Table 2). The results reveal that free-standing ORFs are also an important factor affecting the mitogenome sizes of the Leotiomycetes.

Mitogenomes have an independent evolutionary origin relative to nuclear genomes^31^ and are widely believed to originate from endosymbiotic bacteria^9^. In the course of evolution, most mitochondrial genes of eukaryotes have been transferred to the nuclear genome^32,33^, a process that has been frequent, sporadic, and episodic^32^. The transfer of nucleic acids from the mitochondrion to the nucleus is an ongoing process in most eukaryotes, resulting in the transfer of functional genes^32,34^. The presence of genes in mitochondria has its advantages, such as the production of hydrophobic proteins in mitochondria to avoid long-distance transport from the nucleus, and the maintenance of mitochondrial structure^35,36^. The mitogenome of *S. auriculariicola* retains all 15 typical protein-coding genes (a*tp6, atp8, atp9, cob, cox1, cox2, cox3, nad1, nad2, nad3, nad4, nad4L, nad5, nad6*, and *rps3*) for energy metabolism and transcriptional regulation. However, four species of *Pseudogymnoascus* have lost the mitochondrial *atp9* gene. In addition, the *S. auriculariicola* mitogenome has 31 intronic ORFs and 10 independent ORFs. Most of these ORFs are homing endonuclease genes (25 LAGLIDADG, 11 GIY-YIG), but there are five ORFs with unknown functions. ORFs have been widely found in fungal mitogenomes, though their origin and function have not been elucidated^37^. More studies on the mitogenomes of *S. auriculariicola* and related species are needed to recover the exact functions of these ORF genes.

tRNA genes are important nexus molecules between mRNAs and protein, and are essential for translation^38^. The *S. auriculariicola* mitogenome contains 30 tRNAs, most of which are clustered around *rnl*, *nad6*, and *atp9*. This is similar to *S. borealis*, *P. subalpina*, and *G. lozoyensis*, which also belong to the Leotiomycetes. The number of mitochondrial tRNAs of Leotiomycetes ranges from 27 to 35, which is higher than that of most fungi with 22–26 tRNAs^19,39,40^. These tRNA genes can adequately satisfy the need to decode and predict all of the codons in mitochondrial ORFs, thus reducing the need for tRNAs to enter mitochondria from the cytoplasm^41^. All of the tRNAs in the mitogenome of *S. auriculariicola* have distinctive primary and secondary structures, of which 27 have base mutations and mismatches, with all of them being G-U mismatches. In addition to G-U mismatches, C-U and A-C mismatches also exist in other eukaryotic mitogenomes^42^. Mitochondrial tRNA mutations have been demonstrated to be associated with metabolism and various diseases^43,44^. However, little research has been done on tRNA mutations in fungal mitogenomes. Further studies are needed to investigate the effects of mitochondrial tRNA mutations on the growth and pathogenicity of pathogenic fungi.

The GC content of mitogenomes varies among organisms and is thought to be influenced by mutation bias, selection, and reconstruction-related DNA repair bias^45^. The GC content of the *S. auriculariicola* mitogenome is lower than that of all of the Leotiomycetes except *C. variabilis*, suggesting that the *S. auriculariicola* mitogenome has undergone a large number of variations during its evolution. In addition, the *S. auriculariicola* mitogenome has a uniquely positive AT skew, while the AT skews are negative in the mitogenomes of other Leotiomycetes. According to the second parity rule, as long as there is no mutation or selection bias, each base in the complementary DNA strand exists at approximately equal frequencies^46^. The presence of AT or GC skews on the same DNA strand from different species indicated that mitogenomes of different species underwent different mutations or environmental selection. Compared with other Leotiomycetes, the *S. auriculariicola* mitogenome has a unique A-over-T situation, indicating that it had undergone a unique genetic mutations or evolutionary selection.

Because all of the mitochondria originate from common ancestors, mitochondrial gene rearrangements have been widely used to study the origin and evolution of eukaryotes. Plant and animal mitogenomes have been extensively studied with regard to gene rearrangement, and several models have been established to explain its causes^47,48^. In recent years, there have been many studies on mitochondrial gene rearrangement in fungi^39,49^, which exists even within the same genus^6^. Andrey *et al*.^20^ compared the mitochondrial gene recombination of seven fungi in the Helotiales order and found that the mitochondrial genes can be divided into two main groups. In the present study, we analyzed the mitochondrial gene rearrangement in 16 Leotiomycetes fungi, including *S. auriculariicola*, and divided them into 5 distinct groups. Further, large-scale rearrangements of mitochondrial genes in the Leotiomycetes were confirmed by synteny analysis, indicating that the mitochondrial genes of Leotiomycetes have undergone different evolutionary patterns.

Mitochondrial genomes are widely used in fungal phylogenetic analysis due to their useful and informative markers^50^. Because many fungi are very similar in morphology and difficult to distinguish, fungal classification is easily confused. Phylogenetic and taxonomic analyses of fungi therefore need to be combined with molecular markers, and those of the mitochondrial genome could to be a good complement. In the present study, we constructed phylogenetic trees with 15 protein-coding genes genes. The high support rate indicated that these mitochondrial gene data can be used as reliable molecular markers. However, most single-gene phylogenetic trees exhibited different branches of evolution, and some even failed to distinguish fungi of different classes. This is due to insufficient evolutionary signals provided by individual genes^51^. However, the single-gene evolutionary tree of the *nad4* gene is consistent with that of combined mitochondrial gene set, suggesting that the *nad4* gene can be used as a potential molecular marker for evaluating the taxonomy of Leotiomycetes fungi. The *nad4* gene had relatively conserved gene length (ranging from 1464 bp to 1485 bp) and intron number (zero or one) between different Leotiomycete species. In addition, the *nad4* gene may be subjected to low oxidative stress in mitochondria compared with other mitochondrial genes, which leads to different selective pressures on *nad4* gene. All of these promote nad4 to become a potential molecular marker to analyze phylogenetic relationships of Leotiomycete species.

In conclusion, this study enriches the mitochondrial database of Leotiomycetes fungi and fills in a gap for the mitogenomes of the *Scytalidium* genus. The gene content, structure, gene rearrangement, and phylogenetic analysis of the *S. auriculariicola* mitogenome will provide a basis for population genetics, taxonomy, and evolutionary biology of the Leotiomycetes and related groups.

## Materials and Methods

### Sampling and DNA extraction

The symptomatic mycelium of the pathogen of slippery scar from *A. polytricha* was collected from Jintang, Sichuan Province, China. The isolation of the causative pathogen was conducted according to Peng *et al*.^1^. Suspected fungi were first cultured on PDA medium for 3 days, and then inoculated into cultivation bags with healthy *A. polytricha* mycelia. The inoculated cultivation bags were cultured at 25 °C for 20 days. Then the pathogenic fungi were re-isolated from the cultivation bags with infected *A. polytricha*, which showed the symptoms of slippery scar. The strain was identified as *S. auriculariicola* based on the Koch’s postulates, morphology, and ITS sequences. The mycelium of *S. auriculariicola* was cultured in liquid potato dextrose medium for 4 days and then collected for DNA extraction. Total DNA was extracted from the mycelia using the fungal DNA Kit D3390-00 (Omega Bio-Tek, Norcross, GA, USA) according to the manufacturer’s instructions. The quality of extracted DNA was checked by electrophoresis, and DNA was stored at −20 °C until sequencing. The *S. auriculariicola* strain was stored in Sichuan Academy of Agricultural Sciences (No. SAAS_Sau), and is available from Cheng Chen and Daihua Lu of the Sichuan Academy of Agricultural Sciences, China.

### Sequencing, assembly, and annotation of the mitochondrial genome

Purified DNA was used to construct sequencing libraries following the instructions of the NEBNext Ultra II DNA Library Prep Kit (NEB, Beijing, China). Whole genome shotgun sequencing was performed using an Illumina HiSeq 2500 Platform (Illumina, San Diego, CA, USA). We performed quality control and *de novo* assembly of the mitogenome according to Bi^52^. SPAdes 3.9.0 software^53^ was used for *de novo* assembly of the mitogenome, and the MITObim V1.9 program^54^ was used to fill in the gaps between contigs.

MFannot (http://megasun.bch.umontreal.ca/cgi-bin/mfannot/mfannotInterface.pl) and MITOS^55^ tools were used for mitogenome annotation of *S. auriculariicola*, both of which are based on Genetic Code 4. Uncertain results were adjusted manually by sequence alignments with orthologous genes without intron from the closely related species. The initially annotated protein-coding genes, rRNA, or tRNA genes of *S. auriculariicola* were also modified by alignment with previously published Leotiomycetes mitogenomes. ORFs were functionally annotated by InterProScan software^56^. The tRNAscan-SE 2.0 program was used to predict tRNA genes^57^. Finally, we used the OrganellarGenomeDraw (OGDRAW) tool^58^ to draw a map of the *S. auriculariicola* complete mitogenome.

### Analysis of the mitogenomic organization

We used the Lasergene v7.1 (DNASTAR; http://www.dnastar.com/) tool with default settings to analyze the base composition of the mitogenome of *S. auriculariicola*. Strand asymmetry of the mitogenome was assessed using the following formulas: AT skew = [A − T]/[A + T], and GC skew = [G − C]/[G + C]^59^. We calculated the codon usage using Sequence Manipulation Suite software^60^ based on genetic code 4. We compared the arrangement of genes in *S. auriculariicola* with those of other published Leotiomycetes species. Genomic synteny analysis of mitogenomes from six representative species within the Leotiomycetes was conducted with Mauve v2.4.0^61^.

### Repetitive elements analysis

We searched the entire mitogenome of *S. auriculariicola* by BLASTn searches against itself using Circoletto^62^ (http://tools.bat.infspire.org/circoletto/) with an E-value of <10^−10^, aiming to identify large intragenomic replications of sequences and interspersed repeats. The Tandem Repeats Finder^63^ (http://tandem.bu.edu/trf/trf.advanced.sub-mit.html) with default settings was used to analyze the tandem repeats. We searched for repeated sequences including forward, reverse, complementary, and reverse complementary sequences in *S. auriculariicola* using the REPuter^64^ tool with E-values <10^−5^.

### Phylogenetic analysis

For the phylogenetic analysis, we constructed a phylogenetic tree based on 15 common mitochondrial genes from *S. auriculariicola* and other 15 species in Leotiomycetes, 8 species in Dothideomycetes, 11 species in Eurotiomycetes, and 3 species in Sordariomycetes (outgroup). The MAFFT algorithm within the TranslatorX online platform^65^ was used to align the 15 conserved protein-coding genes. The Sequence Matrix 1.7.8 program^66^ was used to combine the individual genes into a combined matrix. We used the Modelgenerator v851^67^ tool to determine the best-fit evolutionary model for the phylogenetic analysis.

The Bayesian inference (BI) method was used for phylogenetic analysis based on the combined gene dataset with the MrBayes 3.2.6^68^ program. Two independent runs were performed for 2 × 10^6^ generations sampling per 100 generations. Each run was sampled every 100 generations. Stationarity was assumed to have been reached when the estimated sample size (ESS) was >100, and the potential scale reduction factor (PSRF) approached 1.0. After the analysis was stable, the first 25% of the yielded trees were discarded as burn-in, and a 50% majority-rule consensus tree with posterior probability (PP) values was generated from the remaining trees. In order to compare mitochodrial phylogeny with nuclear multi-locus phylogeny, we downloaded internal transcribed spacer (*ITS*), RNA polymerase II second largest subunit (*RPB2*), translation elongation factor-1 alpha (*EF1-α*) and beta-tubulin (*β-TUB*) genes of 38 species from the NCBI database. Phylogenetic trees were constructed using the same method as mitochondrial genes. We also used the BI method to analyze the phylogenetic relationships of *S. auriculariicola* and related species using individual mitochondrial genes (15 core protein-coding genes); the purpose of which is to test whether these genes were useful as molecular markers for the phylogenetic analysis of Leotiomycetes species.

## Supplementary information

Supplementary information

Supplementary information

Supplementary information

## Data Availability

The newly sequenced *S. auriculariicola* mitogenome was deposited to the GenBank database as accession number MK111108.
