# Supplementary figures and images for: Characterization of the mitochondrial genome of the pathogenic fungus Scytalidium auriculariicola (Leotiomycetes) and insights into its phylogenetics
Source: Sci Rep. 2019 Nov 25;9:17447. doi: 10.1038/s41598-019-53941-5 (PMC6877775; doi:10.1038/s41598-019-53941-5)

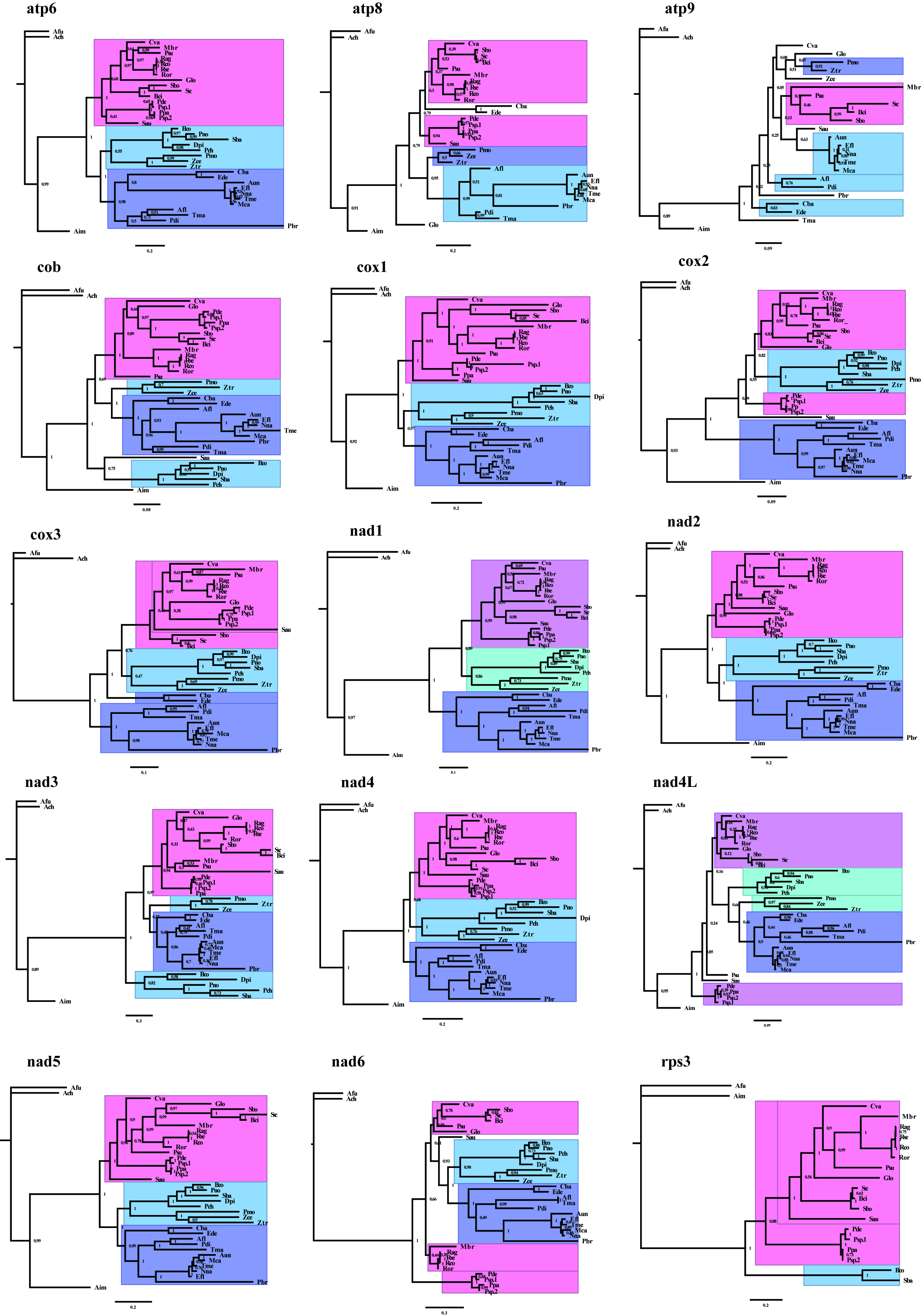

Supplement: Supplementary file 1 — Supplementary information [file 41598_2019_53941_MOESM1_ESM.tif]

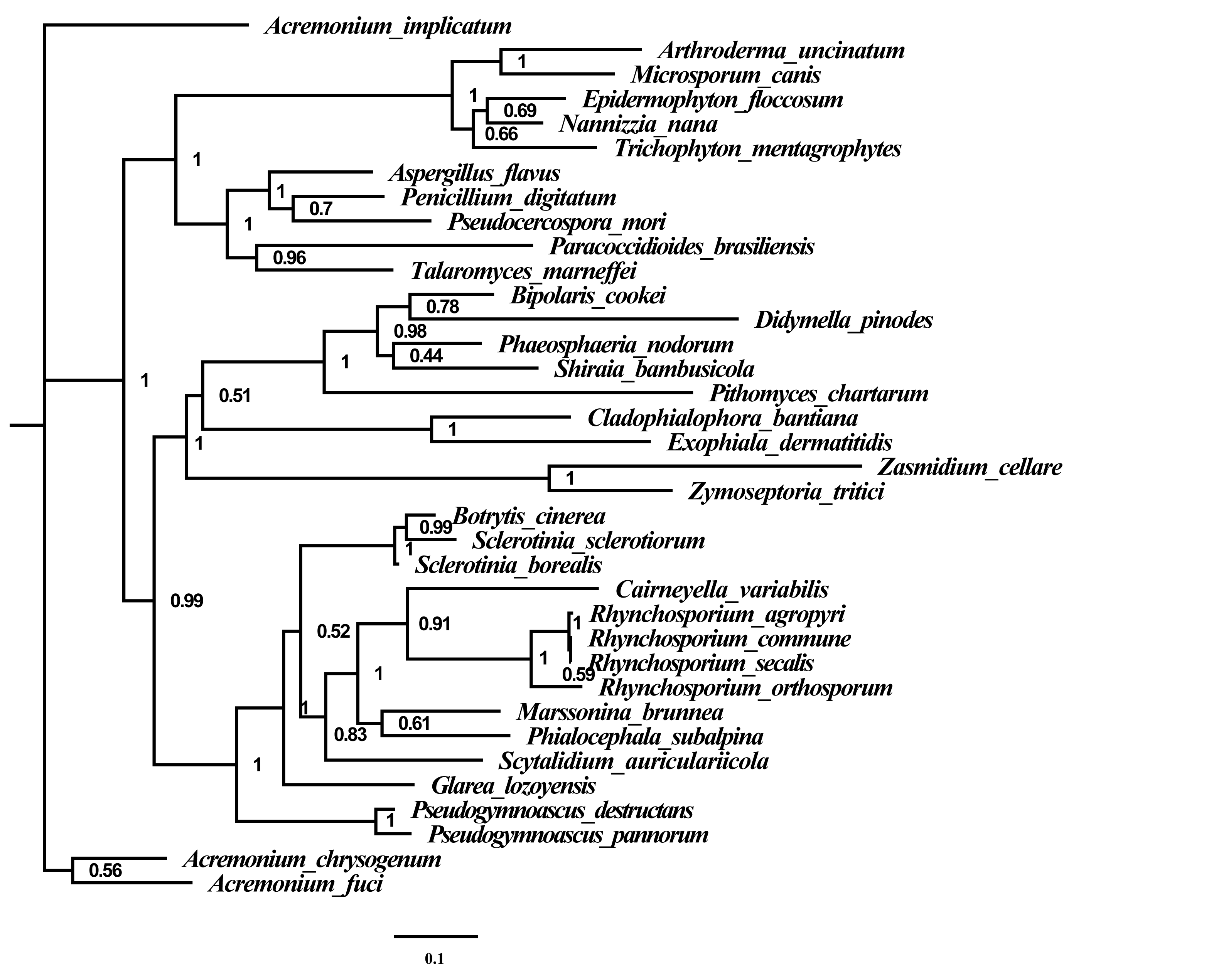

Supplement: Supplementary file 2 — Supplementary information [file 41598_2019_53941_MOESM2_ESM.tif]
